# Scaling plant hydraulic traits to predict ecosystem fluxes under drought

**DOI:** 10.1111/nph.71114

**Published:** 2026-03-19

**Authors:** Yanlan Liu

**Affiliations:** ^1^ Department of Geography University of California Los Angeles CA 90095 USA

**Keywords:** drought, ecosystem diversity, land surface modeling, model‐data integration, plant hydraulics, scaling, traits

## Abstract

Expanding plant hydraulic trait measurements and advances in hydraulic modeling have improved mechanistic predictions of water–carbon fluxes under drought. However, mismatches between individual‐scale traits and ecosystem‐scale model representations introduce prediction uncertainties and obscure how drought impacts propagate across scales. This synthesis identifies four key sources of scale‐induced uncertainty: trait variability, Jensen's inequality, within‐community processes, and compensation errors. I review current efforts to bridge these gaps using both forward modeling and inversion approaches and highlight emerging opportunities from improved characterization of trait variability, mechanistic models that represent community diversity and interactions, and multi‐scale observational networks. Leveraging these opportunities will elucidate the mechanistic propagation of drought impacts across scales and improve predictions of ecosystem flux responses to future droughts.

**Table:** 

	Contents	
	Summary	2078
I.	Introduction	2078
II.	Gaps in using individual‐scale traits to predict ecosystem‐scale fluxes	2079
III.	Scaling hydraulic traits: the forward approach	2081
IV.	Scaling hydraulic traits: the inversion approach	2082
V.	Conclusion and paths forward	2082
	Acknowledgements	2083
	References	2083

## I. Introduction

**Fig. 1:**
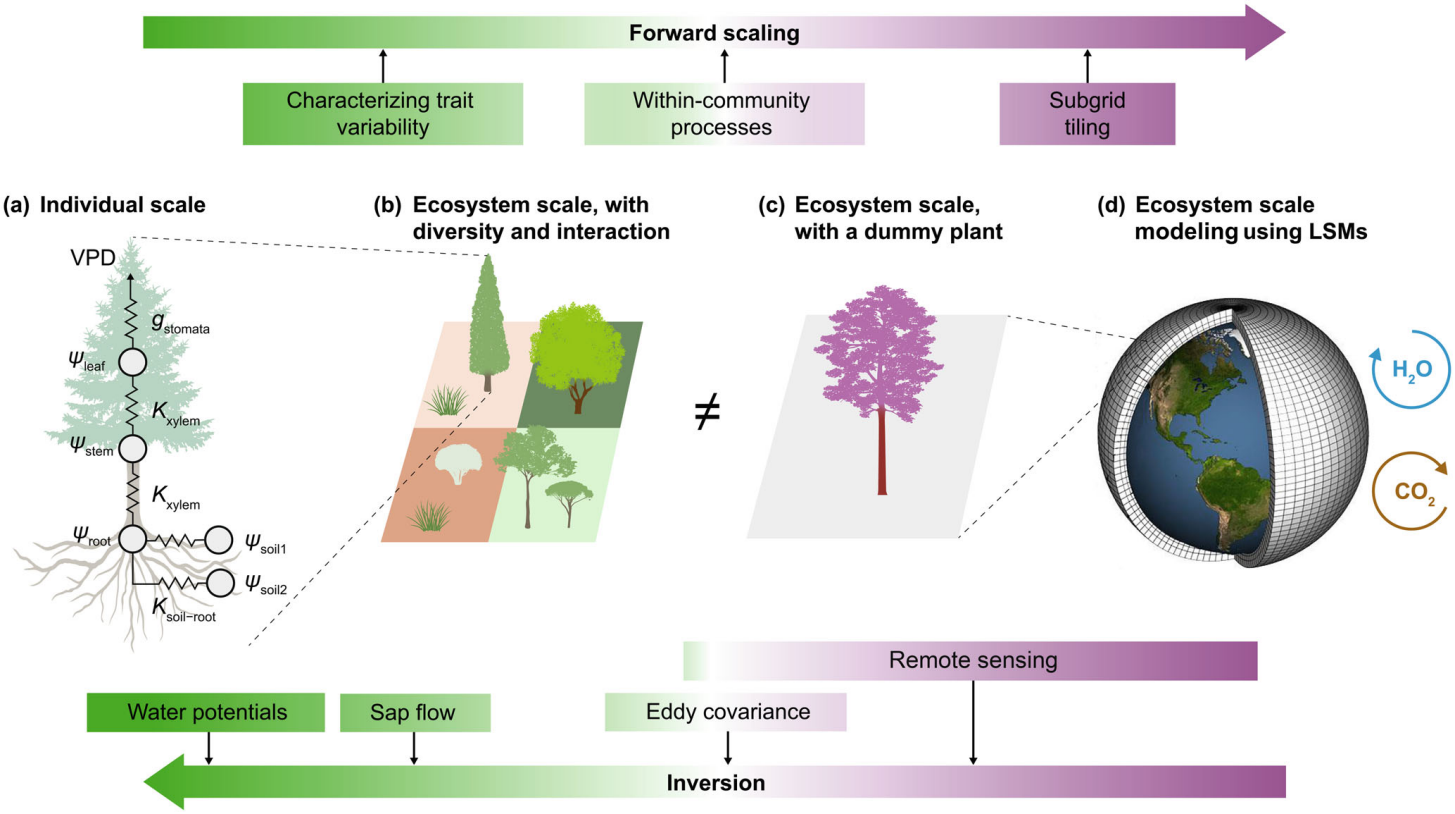
Conceptual diagram illustrating plant hydraulics and the scaling from individual plants to ecosystems, framed by forward and inversion approaches to bridge scale gaps. (a) The basic scheme of plant hydraulics, where plant hydraulic traits regulate hydraulic conductivities ($K_{\text{soil}-\text{root}},K_{\text{xylem}}$), water potentials ($\psi_{\text{root}}$, $\psi_{\text{stem}}$, and $\psi_{\text{leaf}}$), and stomatal conductance ($g_{\text{stomata}}$), which collectively control transpiration and productivity under given soil water potentials ($\psi_{\text{soil}1}$, $\psi_{\text{soil}2}$) and vapor pressure deficit (VPD). (b) Ecosystem with co‐existing species and size classes, where drought responses emerge from individual‐scale hydraulics and interactive within‐community processes. (c) A simplified ecosystem representation with a dummy plant approximating ecosystem‐scale responses, as commonly used in (d) land surface models (LSMs) to model large‐scale water and carbon fluxes. Emerging opportunities to bridge scale gaps are offered by advances in characterizing trait variability, mechanistic modeling of within‐community processes and subgrid tiling, and the expansion of ground‐based observation networks and remote sensing, as detailed in Sections III and IV.

Plant hydraulics determine water movement through plants, mediating ecosystem responses to soil and atmospheric water deficits in drought. These responses form a central hub that have manifested impacts on global hydrological and carbon cycles, and plant survival and ecosystem stability (Torres‐Ruiz *et al*., 2024). At an individual scale, hydraulic responses are shaped by intrinsic hydraulic traits, such as wood specific conductivity, xylem resistance to embolism, shape of pressure‐volume relation, and hydraulically coordinated stomatal traits that control stomatal aperture, such as turgor loss point and nonstressed marginal water use efficiency (Fig. 1a). As key modulators of ecosystem function, hydraulic traits have received much attention over the past decade (Anderegg, 2015; Knighton *et al*., 2024). Expanding global trait measurements at an individual‐scale allow characterization of their variability, offering the premise for predicting drought impacts on ecosystem function (Kattge *et al*., 2020).

Parallel advances in trait‐based modeling have improved predictions and understanding of ecosystem drought responses (Mencuccini *et al*., 2019). In particular, land surface models (LSMs) increasingly incorporate mechanistic representations of plant hydraulics (Kennedy *et al*., 2019; Li *et al*., 2021), replacing empirical soil moisture corrections that often introduce large uncertainties. The mechanistic approaches enable more realistic estimates of photosynthesis, transpiration, and leaf area dynamics under drought (Xu *et al*., 2016; Liu *et al*., 2020; Sabot *et al*., 2022). The modeling progress presents a promising path toward scaling – linking individual trait measurements to large‐scale ecosystem function.

However, spatial scale mismatches between hydraulic trait measurements and model representations pose a major challenge for predicting trait‐regulated ecosystem drought responses (Fig. 1; Mencuccini *et al*., 2019). Ecosystem scale here refers to the spatial unit typically used in LSMs, such as flux tower footprints, subgrid tiles, or gridcells, usually spanning 10^4^–10^9^ m^2^. Although individual‐scale hydraulic traits are measured to represent intrinsic plant properties, ecosystem‐scale model parameters are intended to reflect properties emerging from both constituent individual‐scale hydraulic traits and unresolved (e.g. within‐community) processes (Luo & Schuur, 2020). Directly applying individual‐scale trait measurements as parameters of larger model units can thus introduce substantial errors in flux estimates. Despite broad recognition of this challenge (He *et al*., 2023; Wood *et al*., 2024), limited clarity on the sources of scale‐induced uncertainty hampers mechanistic understanding and effective use of field‐measured traits for ecosystem‐scale predictions. In this review, I unpack key disconnects between individual trait measurements and ecosystem‐scale flux predictions, review existing efforts to bridge the gaps, and outline potential pathways to advance cross‐scale understanding of drought responses and enhance predictions.

## II. Gaps in using individual‐scale traits to predict ecosystem‐scale fluxes

**Fig. 2:**
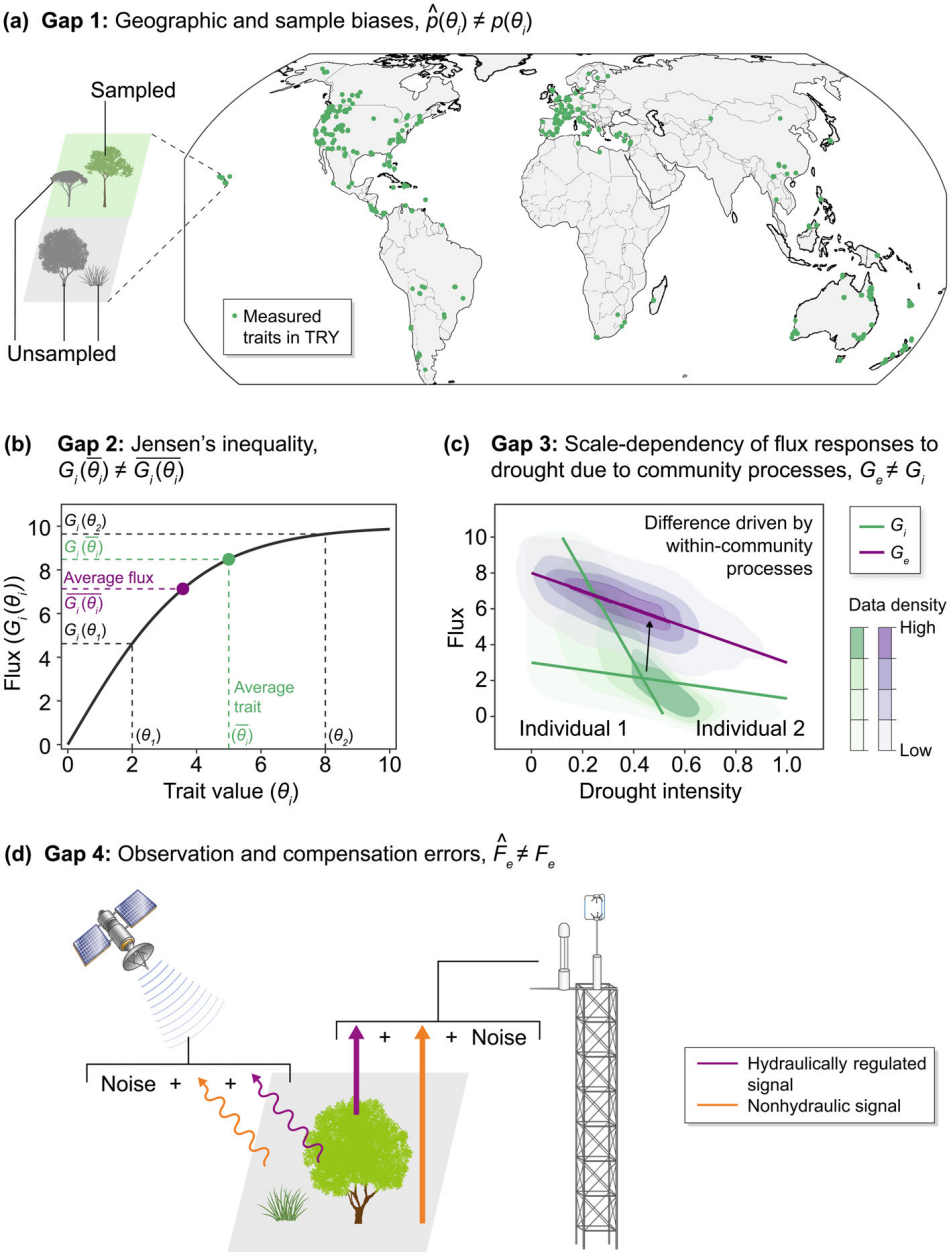
Conceptual illustration of four gaps in scaling hydraulic traits to predict ecosystems‐scale fluxes in drought. (a) Gap 1: trait measurements are unevenly sampled and distributed across regions, leading to mismatches between estimated ($\hat{p}\left(\theta_{i}\right)$) and true ($p\left(\theta_{i}\right)$) trait distributions. Green dots indicate locations with at least one hydraulic trait recorded in the TRY Global Plant Trait Database (Kattge *et al*., 2020), including xylem vulnerability, wood‐specific conductivity, leaf area‐specific conductivity, leaf water capacitance, photosynthetic water use efficiency, and stomatal conductance slope. (b) Gap 2: the average flux ($\bar{G_{i}\left(\theta_{i}\right)}$) differs from the flux calculated from average traits ($G_{i}\left(\bar{\theta_{i}}\right)$) due to nonlinear physiological relationships between traits and fluxes, according to Jensen's inequality. (c) Gap 3: ecosystem‐scale drought responses ($G_{e}$) may diverge from individual‐scale responses ($G_{i}$) determined by physiology alone, as within‐community processes modulate microenvironments. Shaded contours represent the hypothetical variability due to environmental conditions other than drought intensity, with darker colors indicating higher synthetic data density. (d) Gap 4: true ecosystem‐scale fluxes ($F_{e}$, e.g. transpiration and photosynthesis) and plant water content regulated by hydraulics may be inaccurately estimated from observations ($\hat{F_{e}}$), such as eddy covariance and remote sensing, which blend hydraulically regulated signals with nonhydraulic signals and noise. The relative impacts of these four gaps to scale‐induced uncertainties remain to be quantified.

Here, scaling refers to estimating ecosystem‐scale fluxes ($F_{e}$), such as transpiration and productivity, under given climate conditions, based on individual‐scale hydraulic traits ($\theta_{i}$). Formally,

$$F_{e}=G_{e}\left(\Theta_{e}\right)=\int_{\theta_{i}\in \Theta_{e}}G_{i}\left(\theta_{i},E_{i}\left(\Theta_{e}\right)\right)\;p\left(\theta_{i}\right)\;d\theta_{i},$$

where the subscripts *i* and *e* denote individual‐ and ecosystems‐scale quantities, respectively. The ecophysiological function $G_{e}$ translates the collective hydraulic traits of all individuals ($\Theta_{e}=\left\{\theta_{i}\right\}$) to $F_{e}$. This flux can be expressed as the integral of individual fluxes, each governed by individual‐scale physiology ($G_{i}$) depending on $\theta_{i}$ and microenvironment ($E_{i}$, e.g. microclimate and subsurface hydrological conditions), where $\theta_{i}$ follows a joint probabilistic distribution of $p\left(\theta_{i}\right)$, and $E_{i}$ is modulated by $\Theta_{e}$ through plant–plant and plant–environment interactions within the community. From this perspective, four major interconnected gaps emerge (Fig. 2).

### 1. Gap 1: limited representativeness of measured hydraulic traits for ecosystems

Despite expanding global trait databases, the estimated trait distribution $\hat{p}\left(\theta_{i}\right)$ may diverge from the true $p\left(\theta_{i}\right)$. Hydraulic traits are typically measured at the organ level, which may not represent whole‐individual or whole‐ecosystem properties. Even in well‐sampled plots, trait measurements rarely encompass all individuals and are often biased among coexisting species (Sandel *et al*., 2015). Joint measurements of all root‐to‐stomata hydraulic traits remain rare, limiting comprehensive quantification of trait trade‐offs. Geographically, trait measurements are concentrated in temperate regions in the United States and Europe, leaving other temperate and most tropical and boreal zones underrepresented (Fig. 2a; Kattge *et al*., 2020). These biases in hydraulic trait characterization can propagate and undermine ecosystem‐scale flux predictions.

### 2. Gap 2: average traits not sufficiently representing average function

LSMs typically represent vegetation communities using dominant plant functional types (PFTs), summarizing $p\left(\theta_{i}\right)$ as the average traits ($\bar{\theta_{i}}$) for each PFT or as community‐weighted averages based on species abundance. Due to their simplicity, average traits are widely used to estimate ecosystem responses, assuming representative units with average traits can produce ecosystem‐scale fluxes (He *et al*., 2023). However, this assumption departs from Jensen's inequality: the average of a function ($\bar{G_{i}\left(\theta_{i}\right)}$) differs from the function of the average ($G_{i}\left(\bar{\theta_{i}}\right)$), especially under large trait variability and nonlinear hydraulic processes in $G_{i}$ (Fig. 2b). Recent studies show that community‐weighted average traits inadequately explain ecosystem‐scale drought responses, whereas hydraulic trait diversity plays a larger role (Anderegg *et al*., 2018, 2024). Summarizing trait distributions as averages removes variability and trade‐offs that nonlinearly affect hydraulic processes, leading to errors in flux estimates.

### 3. Gap 3: drought responses mediated by within‐community processes

Flux responses to drought are inherently scale‐dependent (Fig. 2c) because of interactions among plants and between plants and the environment. In LSMs, plant hydraulics are typically represented using individual‐scale resistance‐based schemes (Fig. 1a), implicitly equating $G_{e}$ with $G_{i}$. However, $G_{e}$ departs from $G_{i}$ in part because it is additionally mediated by heterogeneous microenvironments ($E_{i}\left(\Theta_{e}\right)$) that emerge from ecosystem assembly and interactions. For example, heterogenous transpiration among individuals reshapes within‐canopy temperature and humidity profiles, altering vapor pressure deficit experienced by plants of different sizes; meanwhile, belowground competition for shared or vertically stratified water pools modify water availability to individual roots, collectively affecting ecosystem‐scale fluxes (Bonan *et al*., 2018; Goulden & Bales, 2019; Mas *et al*., 2024). By reshaping microenvironments, these within‐community processes produce drought sensitivities distinct from those at an individual scale, and if omitted, can bias ecosystem‐scale estimates. Indeed, studies have shown that ecosystem‐scale water use efficiency and evapotranspiration response to drought often diverge from the estimates based on individual‐scale physiology (Medlyn *et al*., 2017; Anderegg *et al*., 2018). The scale dependence of G challenges the generalizability of using individual‐scale traits and physiological functions – even when well constrained – to predict ecosystem‐scale fluxes.

### 4. Gap 4: compensation errors and uncertainty in ecosystem‐scale observations

Observed ecosystem‐scale drought responses, such as fluxes and vegetation water content from eddy‐covariance and remote sensing, provide benchmarks for evaluating scaling approaches (Fisher *et al*., 2020; Liu *et al*., 2020; Konings *et al*., 2021). However, these observations integrate dynamics controlled by plant hydraulics, other nonhydraulic processes, and measurement errors (Fig. 2d). Consequently, models may reproduce observed ecosystem responses for wrong reasons, known as compensation errors. For instance, calibrated nonhydraulic parameters can match ecosystem‐scale evapotranspiration while misrepresenting sensitivities to soil moisture and vapor pressure deficit (Liu *et al*., 2020). Compensation errors may also arise from inaccurate representations of nonhydraulic processes influencing ecosystem fluxes, whether coupled with plant hydraulics (e.g. plant carbon dynamics, energy balance) or relatively independent of it (e.g. soil evaporation, respiration, nutrient cycling; Paschalis *et al*., 2024). Neglecting the complexity in observations and model structure risks misinterpreting the efficacy of scaling efforts.

## III. Scaling hydraulic traits: the forward approach

From a forward or bottom‐up scaling approach (Fig. 1), substantial progress has been made in quantifying trait variability across ecosystems and integrating it into LSMs through innovative parameterization schemes (addressing Gap 1). In parallel, advances in model representations of landscape heterogeneity and community processes have enabled more mechanistic aggregation of physiological responses into ecosystem‐scale functions (addressing Gaps 2 and 3).

Data‐driven methods have been used to impute hydraulic traits of unmeasured species (Knighton *et al*., 2024), enabling broader characterization of trait variability. Modeling studies have shown that more realistically representing trait variability could improve ecosystem‐scale flux estimates under drought. Examples include incorporating trait‐environment and trait–trait relationships into parameterization (Christoffersen *et al*., 2016; Robinett *et al*., 2025), and probabilistically sampling parameters from measurement‐derived $p\left(\theta_{i}\right)$, which better captures trait diversity and trade‐offs than community‐weighted averages (Xu *et al*., 2023). Furthermore, alternative functional classifications based on shared hydraulic behavior, rather than PFTs that omit substantial within‐PFT variation (Anderegg, 2015), can also better represent spatial trait variability without greatly increasing parameterization complexity. These include evolutionary lineages‐based groupings (Anderegg *et al*., 2022) and nondimensional parameter groups that integrate multiple hydraulic traits and environmental variables to reduce dimensionality while retaining characteristic drought responses (Feng *et al*., 2018). Although not yet widely integrated into models, these approaches offer tractable pathways for incorporating field‐derived trait variability to improve ecosystem‐scale predictions.

LSMs provide a powerful platform for integrating trait‐regulated plant hydraulics with other biophysical and biochemical processes and have demonstrated improved predictions of ecosystem‐scale drought responses when plant hydraulics is represented explicitly (Li *et al*., 2021; Sabot *et al*., 2022; Paschalis *et al*., 2024). However, most LSMs aggregate fluxes using subgrid tiles of single PFT, omitting within‐tile community heterogeneity that affect ecosystem‐scale evapotranspiration, water use efficiency, and conservative‐acquisitive tradeoffs (Fig. 1c; Medlyn *et al*., 2017; Anderegg *et al*., 2018, 2024; Fisher & Koven, 2020). Vegetation demographic models address this limitation by representing multiple PFTs and size classes competing for water, light, and nutrients within a tile (Fig. 1b; Fisher & Koven, 2020). This structure enables intrinsic trait parameterization more consistent with individual‐scale measurements, while mechanistically aggregating drought responses from individuals to ecosystems affected by community processes, such as shading, within‐canopy aerodynamics and energy balance, and diverse root water uptake strategies. These ecologically mediated processes generate heterogenous microclimate and belowground competition, nonlinearly regulating ecosystem‐scale drought sensitivity, water use efficiency, and hydraulic vulnerability. Within‐community processes are critical for reproducing realistic biomass regeneration and hydraulically regulated drought deciduousness (Xu *et al*., 2016; Fisher & Koven, 2020), though further research is needed to quantify how they regulate the stability of water‐carbon fluxes under drought.

In summary, recent advances in characterizing trait variability and model representations of within‐community processes have opened promising avenues for forward scaling of measured traits to predict ecosystem drought responses (Fig. 1). However, increased process complexity and parameter dimensionality can amplify model uncertainty (Fisher & Koven, 2020), highlighting the need to develop forward scaling in synergy with inversion approaches.

## IV. Scaling hydraulic traits: the inversion approach

Complementary to forward scaling, the inversion approach derives ecosystem‐scale traits, which represent properties emerging from interactions among $\theta_{i}$, $G_{i}$, and $E_{i}$ of all constituent individuals, enabling ecosystem‐scale models ($G_{e}$) parameterized with these traits to reproduce observed drought responses without explicitly resolving individual‐level and within‐community processes. In most inversion applications, the ecosystem is considered a ‘dummy plant’ (Fig. 1c), allowing physiological principles, such as the resistance‐based scheme (Fig. 1a), to be applied at an ecosystem scale. Individual‐scale measurements of intrinsic traits serve as prior information rather than fixed parameters, enhancing the inference on ecosystem‐scale trait values. This approach has been used to jointly estimate hydraulic traits constrained by sap flow (Lu *et al*., 2022), eddy covariance flux (Liu *et al*., 2020), and remote sensing observations (Liu *et al*., 2021). Inversion approaches have also been used to estimate ecosystem‐scale pressure–volume curves (Konings *et al*., 2021; Wood *et al*., 2024) and stomatal water use efficiency parameters using both offline models (Medlyn *et al*., 2017) and LSMs (Dietze *et al*., 2014; Keetz *et al*., 2025). These inverted traits align with ecosystem‐scale model structures, enabling scale‐consistent estimations of drought responses without added model complexity.

Previous studies have reported discrepancies between inverted ecosystem‐scale traits and individual‐scale measurements (Medlyn *et al*., 2017; Liu *et al*., 2021). While such discrepancies are expected given the Gaps 1–4 (Fig. 2), their major causes and implications remain largely unknown. Inversion approaches mostly bypass Gaps 1 and 2 by directly estimating ecosystem‐scale traits as emergent properties. However, as inversion outcomes rely on model structure and observational constraints, they are susceptible to errors stemming from Gaps 3 and 4. When unresolved nonhydraulic processes or observational errors are inadvertently absorbed into the inverted model parameters as ecosystem‐scale traits, their stability and generalizability may be compromised, especially under novel climate settings.

Studies explicitly targeting Gaps 3 and 4 remain rare. Assessing the impact of Gap 3 on inversion‐based scaling requires testing model configurations with varying complexity in individual and community processes, such as using experimental designs similar to those in carbon cycle modeling (Famiglietti *et al*., 2021). Such experiments can identify hydraulic traits that are mostly model structure‐agnostic, and key nonhydraulic processes and traits that, when jointly constrained, enhance the stability and generalizability of inferred ecosystem‐scale hydraulic traits. Furthermore, inversions likely benefit from incorporating multiple observables that reflect hydraulic dynamics and interacting nonhydraulic processes, ideally across spatial scales (Dietze *et al*., 2014; Fig. 1). This strategy mitigates Gap 4‐induced uncertainties, where reliance on a single observable risks biased trait inference, while more robustly constraining cross‐scale propagation of drought responses. Research in this direction becomes increasingly feasible, given expanding ground‐based networks measuring water potentials (Restrepo‐Acevedo *et al*., 2024), sap flow (Poyatos *et al*., 2016), eddy covariance (Pastorello *et al*., 2020), and emerging remote sensing products that better match field scales, such as high‐resolution evapotranspiration (Fisher *et al*., 2020) and vegetation water content based on global navigation satellite systems (GNSS; Feldman, 2024).

## V. Conclusion and paths forward

Scaling hydraulic traits to predict ecosystem fluxes under drought remains challenging due to difficulties in characterizing trait variability and nonlinear processes that span from individual plants to ecosystems. Although both forward and inversion approaches have contributed to bridging parts of the gaps, they inevitably rely on assumptions to span the remainder. These assumptions have enabled foundational advances under limited data and computational capacity, but now warrant revisiting given recent advances in observational and modeling capabilities.

A critical next step is to quantify the relative and potentially interactive impacts of Gaps 1–4 on scale‐induced uncertainties in drought responses across biomes. Combining observations with numerical experiments provides a tractable pathway to prioritize future scaling efforts. This can be accomplished by comparing observations with modeled drought responses across (i) parameterization schemes with different levels of variability in traits or multi‐trait groups dominating drought responses (Gaps 1 and 2); (ii) flexible model structures of increasing complexity that resolve processes from individuals to ecosystems (Gap 3); and (iii) alternative configurations of observational constraints on hydraulic and interacting nonhydraulic processes across scales (Gap 4). Reporting both consistencies and inconsistencies across scales and between forward and inversion approaches can illuminate where and why gaps persist. In both approaches, aligning model complexity with data availability for processes across scales is essential to alleviate overfitting or compensatory oversimplification (Feng, 2020). Although model development has often outpaced data availability (Dietze *et al*., 2014), intensively monitored sites with concurrent observations of hydraulic traits and multi‐scale drought responses could offer valuable testbeds. For example, when paired with observations, demographically enabled LSMs and their reduced‐complexity counterparts without explicit ecological assembly provide promising tools for testing how trait diversity and community processes shape ecosystem drought responses. These efforts likely reveal quantitative links between individual‐scale hydraulic traits as intrinsic properties and their emergent ecosystem‐scale expressions. Such investigations not only clarify the extent to which a representative ‘dummy plant’ can capture ecosystem‐scale drought responses (Wood *et al*., 2024), but also provide mechanistic insights into how drought impacts are regulated by ecosystems ‘of the most various kinds and sizes’ (Tansley, 1935).

## Competing interests

None declared.

## Disclaimer

The New Phytologist Foundation remains neutral with regard to jurisdictional claims in maps and in any institutional affiliations.
